# Entropy Based Fatigue, Fracture, Failure Prediction and Structural Health Monitoring

**DOI:** 10.3390/e22101178

**Published:** 2020-10-19

**Authors:** Cemal Basaran

**Affiliations:** Department of Civil Structural and Environmental Engineering, University at Buffalo, SUNY, New York, NY 14260, USA; cjb@buffalo.edu

This special issue is dedicated to entropy-based fatigue, fracture, failure prediction and structural health monitoring. The unification of laws of thermodynamics and Newtonian mechanics has been a pursuit of many scientists since the mid-19th century. Distinguished scientists from around the world who contributed to this special issue all show that unification of Newtonian mechanics with thermodynamics using entropy as a link eliminates the need for phenomenological continuum mechanics, where the second law of thermodynamics is usually imposed only as an external constraint, but is not satisfied at the material level, because derivative of displacement with respect to entropy is assumed to be zero. For example, the theory of elasticity assumes that there is no entropy generation at the material level. As a result, everything is reversible, which violates the second law of thermodynamics.

Group from Indian Institute of Technology Madras and University at Buffalo used unified mechanics theory for low cycle fatigue life prediction of Ti-6Al-4V alloys. Bin Jamal et al. [1] show that using unified mechanics theory fatigue life can be predicted using physics, rather than using the empirical curve fitting models. This is also the first peer-reviewed paper in literature to publish the laws of Newton and laws of thermodynamics in unified form at ab-initio level. The second law of unified mechanics theory is given by [1,2]

(1) $$F=m\frac{d\left[v\left(1-\Phi \right)\right]}{dt}$$

where Φ is the Thermodynamic State Index (TSI), a linearly independent axis in addition to Newtonian space-time axes, that can have values between zero and one.

Scientists from Belarus State University contributed a noteworthy paper with their recent advances on mechanothermodynamics, which is essentially a theory almost identical to the unified mechanics theory. They both use entropy generation rate for degradation and unification of Newtonian mechanics and thermodynamics laws. Sosnovskiy and Sherbakov [3] formulate the main principles of the physical discipline of mechanothermodynamics that unites Newtonian mechanics and thermodynamics. Authors state that mechanothermodynamics combines two branches of physics, mechanics and thermodynamics, to take a fresh look at the evolution of complex systems. The analysis of more than 600 experimental results on polymers and metals are used for determining a unified mechanothermodynamics function of limiting states. They are also known as Fatigue Fracture Entropy (FFE) states.

A Purdue University group contributed their outstanding work on using maximum entropy models for fatigue damage in metals with application to low-cycle fatigue of aluminum 2024-T351. Young and Subbarayan [4] propose using the cumulative distribution functions derived from maximum entropy formalisms, utilizing thermodynamic entropy as a measure of damage to fit the low-cycle fatigue data of metals. The thermodynamic entropy is measured from hysteresis loops of cyclic tension–compression fatigue tests on aluminum 2024-T351. The plastic dissipation per cyclic reversal is estimated from Ramberg–Osgood constitutive model fits to the hysteresis loops and correlated to experimentally-measured average damage per reversal. The proposed model predicts fatigue life more accurately and consistently than several traditional models, including the Weibull distribution function and the Coffin–Manson relation. The formalism is founded on treating the failure process as a consequence of the increase in the entropy of the material due to plastic deformation. This argument leads to using inelastic dissipation as the independent variable (which provides the coordinate along TSI) for predicting low-cycle fatigue damage, rather than the more commonly used plastic strain. The entropy of the microstructural state of the material is modeled by statistical cumulative distribution functions, following examples in recent literature. They demonstrate the utility of a broader class of maximum entropy statistical distributions, including the truncated exponential and the truncated normal distribution. Authors show that not only are these functions demonstrated to have the necessary qualitative features to model damage, but they are also shown to capture the random nature of damage processes with greater fidelity.

University of Maryland, College Park scientists contributed an excellent study on measures of entropy to characterize fatigue damage in metallic materials. Yun and Modarres [5] show that Fatigue Fracture Entropy (FFE) is a material property independent of geometry or loading. This paper presents the entropic damage indicators for metallic material fatigue processes obtained from three associated energy dissipation sources. Authors state that, entropy, the measure of disorder and uncertainty, introduced from the second law of thermodynamics, has emerged as a fundamental and promising metric to characterize all mechanistic degradation phenomena and their interactions. Entropy has already been used as a fundamental and scale-independent metric to predict damage and failure. In this paper, three entropic-based metrics are examined and demonstrated for application to fatigue damage. Authors collected experimental data on energy dissipations associated with fatigue damage, in the forms of mechanical, thermal, and acoustic emission (AE) energies, and estimated and correlated the corresponding entropy generations with the observed fatigue damages in metallic materials. Three entropic theorems—thermodynamics, information, and statistical mechanics—support approaches used to estimate the entropic-based fatigue damage. Authors show that classical thermodynamic entropy provided a reasonably constant level of entropic endurance to fatigue failure. Finally, they indicate that an extension of the relationship between thermodynamic entropy and Jeffreys divergence from molecular-scale to macro-scale applications in fatigue failure resulted in an empirically-based pseudo-Boltzmann constant equivalent to the Boltzmann constant.

University of Texas at Austin researchers contributed an excellent paper on degradation-entropy generation methodology for system and process characterization and failure analysis. Osara and Bryant [6] formulated a new fatigue life predictor based on ab initio irreversible thermodynamics. The method combines the first and second laws of thermodynamics with the Helmholtz free energy, then applies the result to the degradation-entropy-generation relation to relate a desired fatigue measure—stress, strain, cycles or time to failure—to the loads, materials and environmental conditions (including temperature and heat) via the irreversible entropies generated by the dissipative processes that degrade the fatigued material. The formulations are then verified with fatigue data from the literature, for a steel shaft under bending and torsion.

Scientists from Northwestern Polytechnical University and Xi’an University of Architecture and Technology contributed an exceptional study titled an entropy-based failure prediction model for the creep and fatigue of metallic materials. Wang and Yao [7] state that it is well accepted that the second law of thermodynamics describes an irreversible process, which can be reflected by the entropy increase. Irreversible creep and fatigue damage can also be represented by a gradually increasing damage parameter. In the current study, an entropy-based failure prediction model for creep and fatigue is proposed based on the Boltzmann probabilistic entropy theory and continuum damage mechanics. A new method to determine the entropy increment rate for creep and fatigue processes is proposed. The relationship between entropy increase rate during creep process and normalized creep failure time is developed and compared with the experimental results. An entropy-based model is developed to predict the change of creep strain during the damage process. Experimental results of metals and alloys with different stresses and at different temperatures are utilized to verify their model. It shows that the theoretical predictions agree well with experimental data.

Universiti Kebangsaan Malaysia group, contributed a great study on prediction of fatigue crack growth rate based on entropy generation. Idris et al. [8] present the assessment of fatigue crack growth rate for dual-phase steel under spectrum loading based on entropy generation. According to the second law of thermodynamics, fatigue crack growth is related to entropy gain because of its irreversibility. In this work, the temperature evolution and crack length were simultaneously measured during fatigue crack growth tests until failure to ensure the validity of the assessment. Results indicate a significant correlation between fatigue crack growth rate and entropy. This relationship is the basis in developing a model that can determine the characteristics of fatigue crack growth rates, particularly under spectrum loading. Predictive results showed that the proposed model can accurately predict the fatigue crack growth rate under spectrum loading in all cases. The root mean square error in all cases is 10^−7^ m/cycle. In conclusion, they prove that entropy generation can accurately predict the fatigue crack growth rate of dual-phase steels under spectrum loading.

Researchers from Beihang University and Beijing Aeronautical Science & Technology Research Institute contributed a very interesting study on using copula entropy for quantifying dependence among multiple degradation processes. Sun et al. [9] studied multivariate degradation modeling to capture and measure the dependence among multiple features. In order to address this problem, this paper adopts copula entropy, which is a combination of the copula function and information entropy, to measure the dependence among different degradation processes. An engineering case study was utilized to illustrate the effectiveness of the proposed method. The results show that this method is valid for the dependence measurement of multiple degradation processes.

Scientists from Beihang University and North China University of Water Resources and Electric Power contributed an indirectly related paper on intelligent analysis algorithm for satellite health under time-varying and extremely high thermal loads. Li et al. [10] present a dynamic health intelligent evaluation model proposed to analyze the health deterioration of satellites under time-varying and extreme thermal loads. New definitions, such as health degree and failure factor and new topological system considering the reliability relationship, are proposed to characterize the dynamic performance of health deterioration. The dynamic health intelligent evaluation model used the thermal network method (TNM) and fuzzy reasoning to solve the problem of model missing and non-quantization between temperature and failure probability.

Nanjing University of Science and Technology and City University of Hong Kong teams participated with their paper titled effective surface nano-crystallization of Ni_2_FeCoMo_0.5_V_0.2_ medium entropy alloy by rotationally accelerated shot peening. Liang et al. [11] reported the surface nano-crystallization of Ni_2_FeCoMo_0.5_V_0.2_ medium-entropy alloy by rotationally accelerated shot peening (RASP). Transmission electron microscopy analysis revealed that deformation twinning and dislocation activities are responsible for the effective grain refinement of the high-entropy alloy. In order to reveal the effectiveness of surface nano-crystallization on the Ni_2_FeCoMo_0.5_V_0.2_ medium-entropy alloy, a common model material, Ni, is used as a reference.
